# Seasonal variation of rare earth elements in *Taraxacum officinale* as an indicator of changes in urban pollution

**DOI:** 10.1038/s41598-025-15371-4

**Published:** 2025-08-12

**Authors:** Marta Lisiak-Zielińska, Klaudia Borowiak, Anna Budka, Patrycja Mleczek, Arlinda Cakaj, Jolanta Kanclerz, Anetta Hanć

**Affiliations:** 1 Department of Ecology and Environmental Protection, Faculty of Environmental and Mechanical Engineering, Poznań University of Life Sciences, Wojska Polskiego 28, 60-637 Poznań, Poland; 2 Department of Construction and Geoengineering, Faculty of Environmental and Mechanical Engineering, Poznań University of Life Sciences, Wojska Polskiego 28, 60-637 Poznań, Poland; 3 Department of Land Improvement, Environmental Development and Spatial Management, Faculty of Environmental and Mechanical Engineering, Poznań University of Life Sciences, Wojska Polskiego 28, 60-637 Poznań, Poland; 4 Department of Trace Analysis, Faculty of Chemistry, Adam Mickiewicz University, Uniwersytetu Poznańskiego 8, 61-614 Poznań, Poland

**Keywords:** Dandelion, Soil-plant relations, City, Bioaccumulation, Elements’ translocation, Urban ecology, Sustainability

## Abstract

*Taraxacum officinale* has been identified as a potential rare earth elements (REEs) accumulator, making it a promising bioindicator for urban environment. However, the influence of seasonal variation on the bioavailability, transport, and accumulation of REEs in plant tissues remains poorly understood. This knowledge gap is crucial, especially in the context of development of reliable bioindicators for urban pollution and managing urban ecosystems sustainably. The aim of this study was to evaluate seasonal changes in the content and distribution of Sc, Y and 14REEs in soils and dandelion roots and leaves from urban areas. Ten research sites typical for urbanised areas were designated and samples of soils and plants were collected in the spring and autumn season. The assessment of the level of studied elements combined with statistical analysis was performed. Variation in REEs accumulation in soil was found. Due to the lack of heavy industry in the city, the major source of REEs contamination in soils might be related to transportation and local agrotechnical and nutrition treatments. Our research on dandelion showed a decrease in REEs content in roots and leaves during autumn compared to spring. Slightly higher translocation efficiency was observed in autumn, possibly due to the plant’s age and tolerance mechanism.

**Supplementary Information:**

The online version contains supplementary material available at 10.1038/s41598-025-15371-4.

## Introduction

The lanthanides (La, Ce, Pr, Nd, Pm, Sm, Eu, Gd, Tr, Dy, Ho, Er, Tm, Yb and Lu), together with scandium (Sc) and yttrium (Y) are considered as rare earth elements (REEs)^1^. Importance of this elements group is an effect of their increasingly crucial for many technological applications, ranging from cell phones to wind turbines^2^. In recent years, there has been an increased interest in the study of REEs biogeochemistry, including their concentration, distribution and bioavailability in the soil-plant system, as well as remediation processes^1^. Unfortunately, our understanding of REEs behaviour in the environment and various biological aspects remains fragmented.

The total content of rare earth elements in soils is closely associated with the parent material. According to Dołęgowska and Migaszewski^3^, typically exhibiting values ranging from 10 to 100 mg kg^− 1^. The concentration of REEs in soils can vary significantly and is influenced by factors such as physico-chemical properties of soil, microbiological activity in soil solution^4^ but also geological substrate and human activities^5^. In urban areas due to their complex functional and spatial structure as well as intense anthropogenic pressure, the local accumulation of REEs might occur particularly in zones with high traffic density, industrial land use and intensive land exploitation^6^. Generally, higher accumulation of REEs by plants growing on substrates enriched with available forms of these metals is observed^7^. In plants, both cultivated and wild species, the range for REEs content is diversified. These variations are primarily determined by plant species and the REEs concentration in the soil, with accumulation levels often being limited to specific habitat conditions, which complicates the comparison of results^5^. Results of different studies have indicated that the accumulation of REEs in the aboveground organs of plants is lower than that in the belowground organs^8^. However, there are exceptions, not only in terms of plant species, but even for individual rare earth elements^5,9^. For example, the distribution of REEs in plants growing on polluted sites is as follow: leaf > stem > roots^10,11^. The reason for the variation can not only be attributed to the difference of REEs abundance in soils alone, but also to specific differences among plants. Even the same species growing in the same location has shown different uptake abilities^12,13^.

Despite extensive studies of REEs use to practical purposes, little is known about the ability of plants to accumulate rare earth elements during different seasons. Seasonality can significantly influence the bioavailability of these elements as well as their transport and storage in plant tissues. The lack of this information represents a critical knowledge gap, especially in the context of assessing potential environmental risks. Understanding the mechanisms of seasonal REEs accumulation by plants is essential for both ecological studies and the sustainable management of natural resources. The seasonal factor seems to be interest, as knowledge of the biogeochemistry of REEs is essential to further assess their potential environmental impact, human exposure and ecotoxicological effects. Seasonal and spatial variations in rare earth elements have been so far analysed mainly in water and water-related ecosystems, e.g. South Korean rivers and rainwaters^14^, Australian catchment^15^, Spanish drainage in a mining area^16^, or mussel in the Portuguese coast^17^. Seasonal changes in the REEs content of plants represent a small part of the ongoing research. Mainly, these studies have been conducted using *Juncus effusus* L^18,19^.

*Taraxacum officinale* called also “dandelion” is a wild plant, which occurred in a wide range of conditions and can be found in all continents, except for Antarctica. Moreover, is able to grow from the sea level to alpine elevation, tolerating almost every soil type. Nevertheless, it prefers to grow in sites where human interference has taken place: along roads or railways, in parks, gardens or abandoned fields and meadows^20^. Dandelion has demonstrated several characteristics that make it a valuable bioindicator, which include wide range of occurrence in the different climatic zones, ability to adopt to hard environmental conditions and its established effectiveness as a passive bioindicator for other pollutants. Previous investigations found that in comparison to other herbaceous plants commonly found in the urban areas, dandelion represents the widest range of occurrence and the biggest potential for elements accumulation^10^. This is crucial for the presented investigations. Many studies with the use of dandelion, have focused on its function as a bioindicator due to its ability to adapt to adverse environmental conditions. Most of these investigations have involved monitoring the environment for dandelion’s ability to bioaccumulate heavy metals^21,22^, but also rare earth elements^10,23,24^. However, previous research regarding the concentration of various elements in *T. officinale* have brought confusing and inconclusive results^21^ and some studies have indicated a strong seasonal influence on the concentration of elements in dandelions^21,25^. This might be probably related to the seasonal variation in plant growth and phenology, as well as to possibility of their accumulation during the season^25^. It has been confirmed by numerous times that herbaceous plants (not limited to those mentioned) have the potential to accumulate of REEs, especially when they grew near roads or in heavily polluted areas^8,26^. Of course, their ability to accumulate was many times lower than known hyperaccumulators such as e.g. *Phytolacca americana*^27^. However, it should be remembered that plants such as *T. officinale* may be characterized by effective accumulation of many other elements, including toxic heavy metals, which emphasizes their importance in the decontamination of polluted soils^28,29^.

Due to the potential of *T. officinale* to REEs uptake, its bioindication characteristics and especially limited data regarding seasonal differences in the profiles of these elements, the objective of this study was to identify patterns of scandium (Sc), yttrium (Y) and 14REEs accumulation in both plant tissues and soils. This research also focused on understanding the seasonal fluctuations in the distribution of these elements across plant organs, as well as the site-specific variations influenced by anthropogenic pressure and seasonal changes in plant accumulation.

## Materials and methods

### Study area

**Fig. 1:**
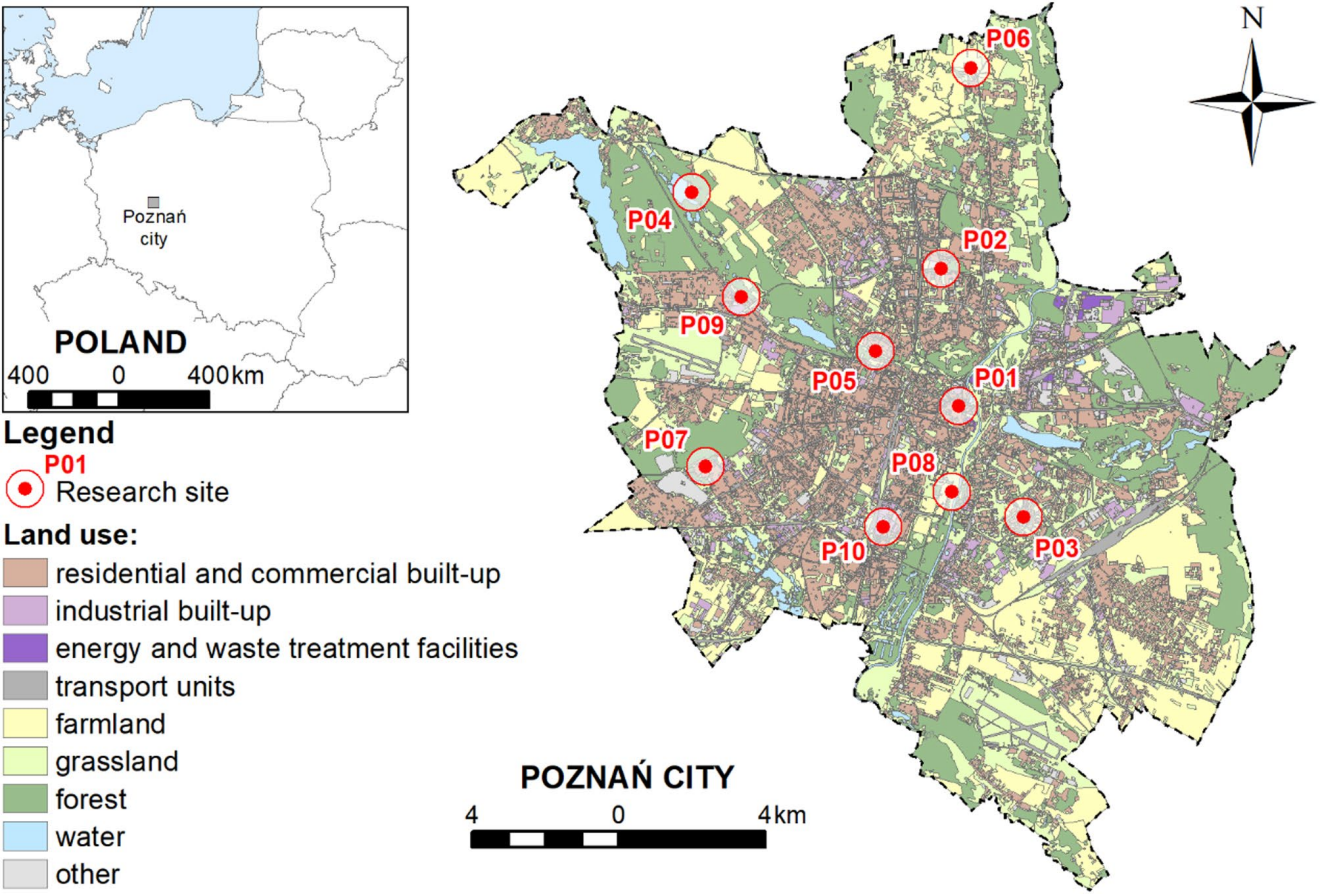
Location of research sites in Poznań city (source: own study based on data from Central Geodetic and Cartographic Resource).

The samples of plants (*Taraxacum officinale*) and soil were collected in Poznań city (Poland), which can represent numerous European cities due to its geographical and climatic conditions as well as the environmental and spatial problems. The analysed city is located in the west part of Poland (52°24′30″ N; 16°56′03″ E) and with an area of approximately 261.91 km^2^ is inhabited by about 545 000 people. Residential areas consist of various types of buildings. The traditional downtown buildings (tenement houses) are located in the centre, while the intensive residential development in the form of blocks of flats is located in the northern, eastern, southern, and south-western parts of the city. Concentration of single-family residential areas occurs in the western and south-eastern parts of the city. Industrial areas are located mainly in the north-eastern and southern parts of the city. There is a landfill, a waste incineration plant, and a power and heating plant in the north of Poznań. However, the most dynamically developing region with an industrial function is located in the eastern part of the city. Service and transportation areas comprise a small part of the city’s area. Both, industrial and services areas, are associated with low environmental impact and are most often commercial centres and logistic centres or local businesses. The agro-food sector and the developing automotive industry are located in the surroundings of the city. A large part of the Poznań area is occupied by arable land, while land under water covers a small area of the city (Fig. 1). The city is characterized by a cool temperate climate. The warmest months, July and August, average temperatures ranging from 18 °C to 23 °C, with occasional peaks exceeding 30 °C. January, the coldest month, typically records average temperatures between − 2°C and 3 °C. Annual precipitation in Poznań averages around 500–600 mm, distributed relatively evenly throughout the year. However, as with temperature, precipitation patterns are also shifting due to global climate change. Recent decades have shown a tendency toward longer dry spells, interrupted by shorter, more intense rainfall events, reflecting increasing weather variability and extreme events^30^.

Most of the research sites were located on loamy sands. Two sites (the areas near main road and railway – P02 and P09, respectively) were located on the sands, and two sites (the high-density and low-density residential areas – P03 and P07, respectively) were located on sandy loams.

### Sample collection

In the city, 10 research sites typical for urbanised areas were designated: the old town (P01), the areas near main road (P02), the high-density residential areas (P03), the areas near Strzeszyńskie lake (P04), park (P05), the rural areas (P06), the low-density residential areas (P07), the areas near Warta river (P08), the areas near railway (P09) and the industrial areas (P10). The selected research sites offered a better representation of the environmental functioning characteristics of numerous European urban areas.

The plant samples (above-ground parts of the dandelion without flowers, roots) and soil samples were collected between 13th and 14th of May for spring season and between 10th and 12th of October for autumn season in 2021. For both seasons, every effort was made to ensure that the weather conditions were similar (collection of samples in rainless weather, after at least 3 days without precipitation). In each research site, five smaller areas were indicated from which at least five samples of plants were collected in the spring and autumn. The harvested dandelions had a similar vegetative phase and standard morphological appearance without visible mechanical damage or disease symptoms. The mean plant high in spring and autumn was 142 ± 31, and 128 ± 25 mm, respectively, while the mean biomass 4.57 ± 1.41 and 4.02 ± 1.25 g, respectively with the percentage share of biomass of roots in whole plants biomass: 32.9% and 32.1%, respectively. Moreover, a total of 0.5 kg of underlying soil samples were collected from a 0–20 cm depth under the plants using a soil auger. After collection, the plant and soil samples were placed in separate clean plastic containers to prevent contamination and were immediately transported to the laboratory. The collected plant material (*Taraxacum officinale*) was deposited in the collection of the Department of Ecology and Environmental Protection at the Poznań University of Life Sciences.

### Sample preparation process and digestion methods

In the laboratory, the plant samples were carefully purified with deionized water using Milli-Q Advantage A10 Water Purification Systems, Merck Millipore (Merck, Darmstadt, Germany) to remove dust and soil particles. After washing, the plant material was separated into leaves and roots. Samples (soil, roots and leaves) were dried at 40 ± 3 ◦C in an electric oven (FD115, Binder, Germany) until they reached a constant weight. Then the plant parts were ground into a fine powder, while the soil samples were homogenized and sieved. Subsequently, approximately 0.2000 g of plant sample and 0.1000 g of soil sample were accurately weighed into Teflon vessels of an Ethos One (Millestone, Italy) pressure digestion system. The samples underwent digestion via two distinct methods. For the plant samples, aliquots were digested using 7 mL of 65% HNO_3_ (Suprapur, Merck, Germany) and 1 mL of 30% H_2_O_2_ (Suprapur, Merck, Germany). These prepared samples were subjected to pre-mineralization for 3 h, after which the vessels were sealed and subjected to pressure digestion at 220 °C with a microwave power of 1500 W for 90 min. For the soil samples, digestion was performed using a mixture of 4 mL of 65% HNO_3_ (Suprapur, Merck, Germany), 2 mL of 67% HCl (Merck KGaA, Darmstadt, Germany), and 2 mL of 48% HBF_4_ (Sigma Aldrich, Missouri, USA) to ensure complete dissolution of all silicates. This digestion process was conducted at 180 °C for 120 min with a microwave power of 1500 W. Post-digestion, the samples were quantitatively transferred into polyethylene tubes and diluted to a final volume of 20 mL using distilled ultrapure water (Direct-Q 3UV Water Purification System, Merck, Germany).

The rare earth elements, scandium and yttrium are significantly affected by polyatomic molecular interferences^31^. Therefore, prior to ICP-MS analysis, the prepared samples were passed through an ion exchange C18 column (ChromaBond, Macherey-Nagel, Germany) loaded with a mixture of ethyl-hexyl-phosphates to eliminate matrix interferences, following the procedures described by Bau et al.^32^ and Zocher et al.^33^. A Tm standard solution spike (PerkinElmer, USA) was added to monitor the recovery of REEs throughout the sample preparation process^32^.

### Quantification methods for rare Earth elements

The concentrations of REEs in plant and soil samples post-digestion were determined using an ICP-MS (Agilent 7700x, Agilent Technologies, Tokyo, Japan) equipped with an Octopole Reaction System (ORS) utilizing helium as a reaction gas to eliminate spectral interferences. Signal drift was corrected using internal standards such as bismuth (Bi) and rhodium (Rh), which have atomic masses that bracket the range of REE masses. Isobaric interferences for each REEs were addressed through appropriate mathematical corrections. The instrument was optimized daily with a tuning solution containing lithium (Li), cobalt (Co), yttrium (Y), cerium (Ce), and thallium (Tl) at a concentration of 10 mg L^− 1^. Quantification was achieved through external calibration, spanning a concentration range from 0.01 mg L^− 1^ to 100 mg L^− 1^ for REEs. Calibration solutions were prepared from a rare earth elements mix for ICP (10 mg L^− 1^ TraceCERT for ICP, Merck, Germany) and a single-element standard of yttrium (1000 mg L^− 1^, Merck, Germany).

### Analytical method performance

To evaluate the precision and trueness of the analytical method, two certified reference materials (CRMs) were utilized: NIST SRM 1570a (Trace Elements in Spinach Leaves) and NIST SRM 1646 (Estuarine Sediments) from the National Institute of Standards and Technology (NIST, Gaithersburg, MD, USA). These CRMs were selected to match the matrix of the plant and soil samples. The CRMs were prepared following the same procedure as described for the plant and soil samples and were subjected to identical treatment at each stage of the measurement process. The repeatability of REEs determination using ICP-MS ranged from 1 to 3.2% RSD (Relative Standard Deviation), while reproducibility of the results was within the range of 2–5% RSD. The recovery rates for NIST SRM 1570a ranged from 92% for lanthanum (La) to 99% for europium (Eu), while for NIST SRM 1646, the recoveries ranged from 85% for lutetium (Lu) to 103% for cerium (Ce).

Basic soil parameters, including pH and electrical conductivity, were also analyzed. Soil samples were dried, homogenized and sieved through a 2 mm mesh sieve. Texture of soil was determined using the combined method^34^. The division into texture groups was made according to the USDA^35^. Soil pH and electrical conductivity (EC) were determined using multiparametric system 3630 IDS (WTW, Germany). The pH was measured in a 1:5 (mass: volume) suspension of soil in 1 mol dm^− 3^ KCl solution, while EC was measured in a 1:5 (mass: volume) suspension of soil in deionized water.

### Contamination, accumulation and translocation factors

Pollution with particular REEs of soil and plant organs (roots and leaves) were estimated using the following four factors: contamination factor (CF), pollution load index (PLI), bioconcentration factor (BCF) and translocation factor (TF).

To assess the level of soil pollution, the contamination factor (CF) was calculated using the Eq. (1):

1 $$\:{\text{C}\text{F}}_{\text{i}}={\text{C}}_{\text{i}}/{\text{C}}_{{\text{n}}_{\text{i}}}$$

where: $\:{C}_{i}$ is the mean content of REEs in soil and $\:{C}_{{n}_{i}}$ is the reference value for REEs^36^. The references value [mg kg^− 1^] were as follows: Sc – 9.1, Y – 22.7, La – 25.9, Ce – 52.2, Pr – 6.02, Nd – 22.4, Sm – 4.28, Eu – 0.851, Gd – 4.20, Tb – 0.638, Dy – 3.58, Ho – 0.716, Er – 2.1, Tm – 0.312, Yb – 2.09 and Lu – 0.307^37^. On the basis of obtained CF values, the degree of pollution can be classified: CF_i_ < 1 – low contamination factor (LCF); 1 ≤ CF_i_ < 3 – moderate contamination factor (MCF); 3 ≤ CF_i_ < 6 – considerable contamination factors (CCF); and CF_i_ ≥ 6 – very high contamination factor (VHCF)^38^.

The pollution load index (PLI) was used to assess the degree of contamination of summarized rare earth elements in soil. The PLI was calculated as follows Eq. (2):

2 $$\:\text{P}\text{L}\text{I}={\left({\text{C}\text{F}}_{1}\times\:{\text{C}\text{F}}_{2}\times\:\dots\:\times\:{\text{C}\text{F}}_{\text{n}}\right)}^{\frac{1}{\text{n}}}$$

where: $\:n$ is the number of REEs and $\:CF$ is contamination factor analysed REEs^39^. PLI was classified according to the following criteria: PLI < 1 – no pollution (NO); 1 ≤ PLI < 2 – moderate pollution (MP); 2 ≤ PLI < 3 – heavy pollution (HP); and PLI ≥ 3 – extremely heavy pollution (EHP)^40^.

To estimate the ability of dandelion to accumulate REEs, the bioconcentration factor (BCF) as a ratio of REEs content in plant samples and REEs content in soil samples^41^ was calculated according to Eq. (3):

3 $$\:\text{B}\text{C}\text{F}=\text{R}\text{E}\text{E}\text{s}\:\text{c}\text{o}\text{n}\text{t}\text{e}\text{n}\text{t}\:\text{i}\text{n}\:\text{p}\text{l}\text{a}\text{n}\text{t}\text{s}\:\left[{\text{m}\text{g}\:\text{k}\text{g}}^{-1}\right]/\text{R}\text{E}\text{E}\text{s}\:\text{c}\text{o}\text{n}\text{t}\text{e}\text{n}\text{t}\:\text{i}\text{n}\:\text{s}\text{o}\text{i}\text{l}\:\left[{\text{m}\text{g}\:\text{k}\text{g}}^{-1}\right]$$

where: BCF value lower than 1 indicates no accumulation in plant biomass, while a value greater than 1 indicates accumulation.

The translocation factor (TF) was used to assess the efficiency of translocation of REEs to aboveground biomass^42^. The TF was calculated as follows Eq. (4):

4 $$\:\text{T}\text{F}=\text{R}\text{E}\text{E}\text{s}\:\text{c}\text{o}\text{n}\text{t}\text{e}\text{n}\text{t}\:\text{i}\text{n}\:\text{l}\text{e}\text{a}\text{v}\text{e}\text{s}\:\left[{\text{m}\text{g}\:\text{k}\text{g}}^{-1}\right]/\text{R}\text{E}\text{E}\text{s}\:\text{c}\text{o}\text{n}\text{t}\text{e}\text{n}\text{t}\:\text{i}\text{n}\:\text{r}\text{o}\text{o}\text{t}\text{s}\:\left[{\text{m}\text{g}\:\text{k}\text{g}}^{-1}\right]$$

where: TF value lower than 1 indicates a lack of efficient translocation in the plant.

### Statistical analysis

To analyze the range of rare earth element content depending on the season, measures of central tendency (mean) and dispersion (standard deviation and range) were calculated. The distributions of the studied elements were additionally described using quartiles to identify asymmetry and detect outliers. A summary of these characteristics is presented in a consolidated table.

The influence of experimental factors on the content of rare earth elements in the studied materials (soil, plant: root, leaf), depending on the season and the site representing different types of land use, was examined using two-way ANOVA. The analysis was conducted separately for each type of material, where the value *y*_*ij*_​ represented the content of each element in *i* seasons (*i* = 1, 2) at *j* different research site types (*j* = 1,…, 10). The effects model in the two-way ANOVA, including the interaction between factors, was as follows (5):

5 $$\:{y}_{ij}={\upmu\:}\:+\:{\alpha\:}_{i}+{\beta\:}_{j}+{\left(\alpha\:\beta\:\right)}_{ij}+{\epsilon\:}_{ij}$$

where: µ – grand mean, *α*_*i*_ – effect of the *i*-th season, *β*_*j*_ – effect of the *j*-th research site type, *(αβ)*_*ij*_ – interaction effect between season and research site type, *ε*_*ij*_ – random error term. The Tukey’s test (a multiple comparison procedure) was used to compare the mean concentrations of rare earth elements across different seasons and site types^43^.

To investigate the correlation structure and the main sources of variability in the concentrations of rare earth elements in the studied materials collected during two seasons, principal component analysis (PCA) was conducted. The analysis included data on REEs content in soil samples and plant organs (roots and leaves) collected during two research seasons: spring and autumn. Due to differing units and ranges, all variables were standardized using Z-score transformation (mean = 0, standard deviation = 1), and the analysis was based on the correlation matrix. For each season and each material type, a biplot was created to illustrated the positions of individual sampling sites in the space of the first and second principal components, along with vectors of the variables (REEs contributions).

## Results

### Soil characteristics

The soil pH ranges from 6.19 to 7.72, with mean values of 7.22 (May) and 7.16 (October). The electric conductivity (EC) of soil extracts varied from 0.06 to 0.19 (mS cm^− 1^), with mean values 0.12 (May) and 0.14 (October) (Supplementary Table S1).

### REEs content in soil and plant organs

The REEs content in soil varied across sites and seasons as reported below: Sc ranged from 1.08 to 3.32 mg kg^−1^ and Y from 3.99 to 16.7 mg kg^−1^. The range of concentrations in soil for 14REEs was as follows: La from 3.72 to 26.0 mg kg^−1^, Ce from 10.6 to 45.9 mg kg^−1^, Pr from 1.97 to 4.37 mg kg^−1^, Nd from 3.55 to 24.7 mg kg^−1^ and Sm from 0.876 to 4.13 mg kg^−1^. The levels of the remaining elements in the soil varied within the following ranges: Eu 0.227–1.66 mg kg^−1^, Gd 1.16–5.18 mg kg^−1^, Tb 0.155–0.767 mg kg^−1^, Dy 0.788–2.83 mg kg^−1^, Ho 0.152–0.559 mg kg^−1^, Er 0.437–1.79 mg kg^−1^, Tm 0.057–0.232 mg kg^−1^, Yb 0.386–1.96 mg kg^−1^, and Lu 0.053–0.229 mg kg^−1^. Overall, REE concentrations were consistently higher in soil than in plant organs in both seasons (Supplementary Table S1).

The ranges of the elements in *T. officinale* for both seasons were recorded accordingly: Sc ranged from 0.122 to 0.587 mg kg^−1^ in roots and from 0.102 to 0.795 mg kg^−1^ in leaves. The Y content varied between 0.299 and 1.65 mg kg^−1^ in roots, and between 0.207 and 2.24 mg kg^−1^ in leaves. The content of the 14REEs in the plants varied within specific ranges. La ranged from 0.309 to 3.82 mg kg^−1^ (roots) and from 0.379 to 3.81 mg kg^−1^ (leaves), while Ce was 0.653–6.27 mg kg^−1^ and 0.002–6.89 mg kg^−1^, respectively. The values for Pr were from 0.075 to 0.704 mg kg^−1^ (roots) and from 0.012to 0.765 mg kg^−1^ (leaves). Nd ranged between 0.286 and 3.09 mg kg^−1^ (roots) and 0.103–2.98 mg kg^−1^ (leaves), while Sm ranged from 0.054 to 0.667 mg kg^−1^ in roots and from 0.042 to 0.561 mg kg^−1^ in leaves. The rest of the determined REEs content in roots and leaves were as follow: Eu 0.014–0.157 and 0.001–0.126 mg kg^−1^, Gd 0.053–0.745 and 0.031–0.593 mg kg^−1^, Tb 0.007–0.073 and 0.002–0.070 mg kg^−1^, Dy 0.033–0.435 and 0.023–0.367 Ho: 0.006–0.060 and 0.001–0.065 mg kg^−1^, Er 0.018–0.158 and 0.011–0.181 mg kg^−1^, Tm 0.002–0.022 and 0.001–0.024 Yb 0.015–0.122 and 0.011–0.163 mg kg^−1^, Lu 0.002–0.020 and 0.001–0.023 mg kg^−1^. REEs content in roots and leaves showed greater seasonal variability in autumn (October) than in spring (May), though interquartile ranges (Q1–Q3) remained comparable between seasons (Supplementary Table S1).

### Contamination, accumulation and translocation factors

The contamination factor (CF) for soil was below 1 for most elements, indicating a low contamination factor (LCF). The analysed factor only for europium slightly exceeded the value of 1 for two research sites – in the park (P05) and in the industrial areas (P10), and was 1.047 and 1.006, respectively. A similar factor value was observed for gadolinium at one research site (P05). For these three values, the index can be classified as a moderate contamination factor (MCF). Due to the low values of the contamination factor the pollution load factor (PLI) was close to zero for all test sites in both seasons. This indicated that there was no contamination (NO) of the soil with rare earth elements (Supplementary Table S2).

**Fig. 2:**
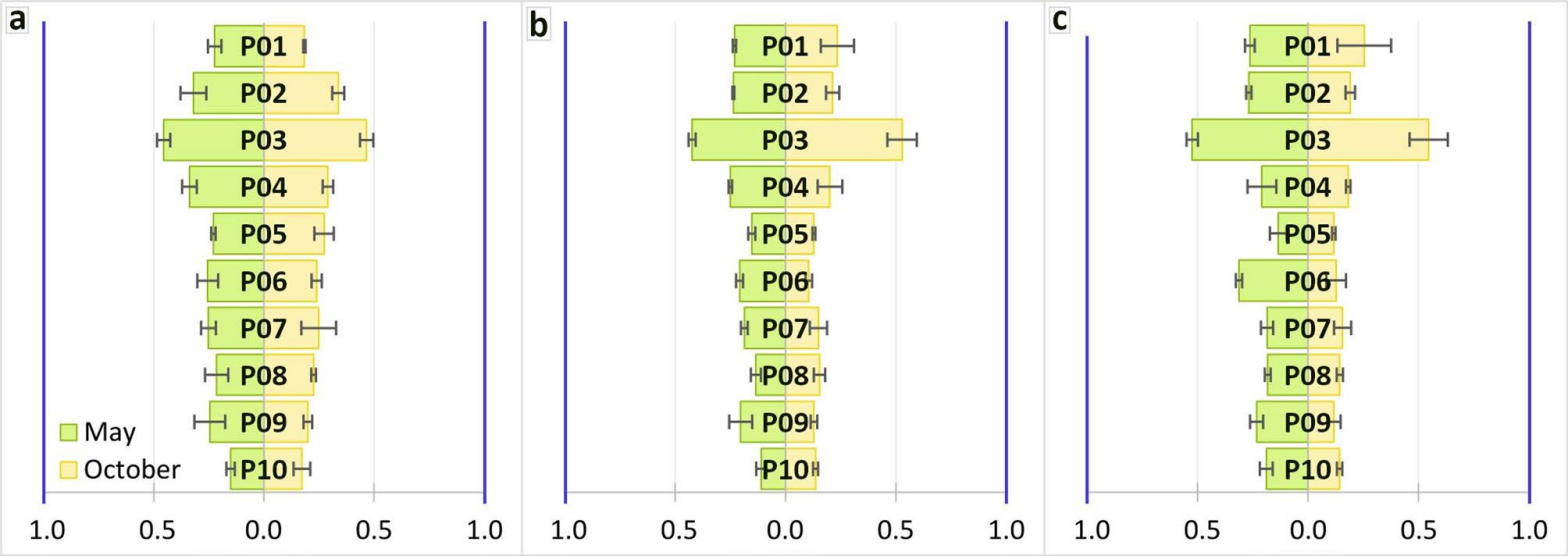
Bioconcentration factor for Sc (**a**), Y (**b**) and calculated sum of 14 REEs (**c**), where the blue line indicted REEs accumulation in plant biomass.

The bioconcentration factor (BCF) for analysed elements did not exceed a value of 1 at any of the research sites, indicating a lack of REEs accumulation in plant biomass. The highest BCF value was observed at the research site representing residential areas (P03). Additionally, BCF values were slightly higher in spring (Fig. 2).

**Fig. 3:**
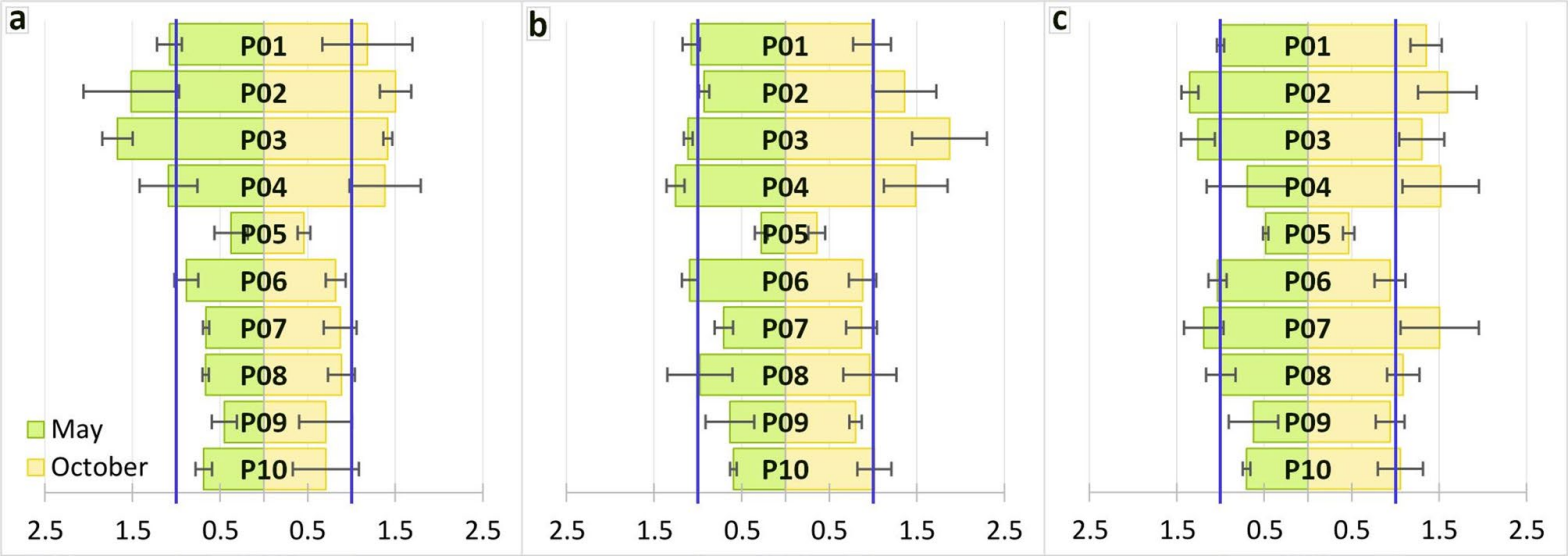
Translocation factor for Sc (**a**), Y (**b**) and calculated sum of 14 REEs (**c**), where the blue line indicted REEs efficient translocation in the plant.

The translocation factor (TF) for the determined elements reached a value above 1 at most research sites, indicating their efficient translocation within the plants. The exception was site located in the park (P05), where none of the elements exceeded the value of 1. In addition, at the site representing industrial areas (P10), all analysed elements had TF values below 1, but only during the spring period (May). A notable trend was also observed whereby the translocation factor for all elements was higher in the autumn period (October) (Fig. 3).

### Assessment of seasonal and site variability

A comparison of the seasonal variation between the determined elements in soil and plants as well as at the research sites was carried out using a two-way analysis of variance with interaction (ANOVA). In the conducted analysis, for most elements, the interaction between season and site was not statistically significant. Therefore, in subsequent stages of the study, only the main effects were considered.

The seasonal differences in REEs accumulation in soil and plants were observed for selected elements. In soil, the content of analyzed elements was generally lower in autumn. However, only the values observed for scandium differed significantly between seasons. Moreover, the content of Sc, Y and 14REEs in dandelion roots differed significantly between spring and autumn. For leaves, seasonal differences were significant for Sc and 14REEs (Table 1).

**Table 1:** Content of individual elements in soil as well as in dandelion roots and leaves in May and October.

Season	Soil			Roots			Leaves		
	Sc	Y	14REEs	Sc	Y	14REEs	Sc	Y	14REEs
F (*p-value)*	5.58 (≤ 0.05*)	2.27 (0.14)	2.48 (0.12)	21.66 (≤ 0.05*)	26.36 (≤ 0.05*)	59.96 (≤ 0.05*)	4.65 (≤ 0.05*)	0.022 (0.88)	17.95 (≤ 0.05*)
May	2.58^a^	7.11	56.7	0.368^a^	0.788^a^	6.99^a^	0.312^a^	0.639	6.39^a^
October	2.46^b^	6.75	53.9	0.309^b^	0.620^b^	4.76^b^	0.291^b^	0.634	5.22^b^

Mean values for season (*n* = 50); identical superscripts (a, b, c …) indicated non-significant differences between means within columns (separately for soil, roots and leaves) according to the post-hoc Tukey’s HSD test.

The content of Sc, Y, and 14REEs in soil, roots, and leaves varied significantly between research sites in both May and October. Sc in soil showed little variation between sites in spring, except for a significantly different value for sites near the main road (P02), and this trend also was observed in autumn. Yttrium content in soil had broader variation in May, with the park site (P05) differing significantly from others in both seasons. Soil content of 14REEs at the research site located in the park (P05) also stood out statistically compared to most other sites. Sites located in the old town (P01) and near the main road (P02) showed similar 14REEs levels but differed from the site in the park (P05), the low-density residential areas (P07) and the industrial area (P10). These site-based patterns remained consistent across both seasons. For the content of Sc, Y and 14REEs in the root of dandelions, the research sites located in the park (P05) but also at the high-density residential areas (P03) were notable different in relation to the other sites. This trend persisted in autumn. Moreover, Sc in roots at research site near the railway (P09) showed elevated values in spring. In leaves, inter-site differences in content of Sc, Y, and 14REEs were also significant. Sc was more evenly distributed than Y and 14REEs, especially in contrast to root distribution. The variation in 14REEs was higher in autumn. The research site located in the high-density residential areas (P03) consistently had the highest elemental content in leaves, while a site located in the industrial area (P10) generally showed the lowest Sc and Y levels. For 14REEs, the lowest values varied by season and location (Table 2).

**Table 2:** Content of sc, Y and 14REEs in soil as well as in dandelion roots and leaves at each research site in May and October.

Research site	Soil			Roots			Leaves		
	Sc	Y	14REEs	Sc	Y	14REEs	Sc	Y	14REEs
May									
F (*p-value)*	7.81 (≤ 0.05*)	27.66 (≤ 0.05*)	30.65 (≤ 0.05*)	8.12 (≤ 0.05*)	151.62 (≤ 0.05*)	13.49 (≤ 0.05*)	81.58 (≤ 0.05*)	37.59 (≤ 0.05*)	19.92 (≤ 0.05*)
P01	2.40^a^	4.90^ef^	38.6^e^	0.255^cd^	0.550^e^	5.10^c^	0.275^cd^	0.586^cd^	5.07^b^
P02	1.68^b^	4.78^f^	38.3^e^	0.213^bd^	0.588^e^	4.37^c^	0.290^cd^	0.544^cde^	5.19^b^
P03	2.71^a^	5.97^def^	52.9^cde^	0.462^a^	1.20^b^	12.5^a^	0.767^a^	1.32^a^	15.3^a^
P04	2.52^a^	6.75^cde^	51.4^cde^	0.424^ab^	0.750^d^	6.30^c^	0.431^b^	0.940^b^	4.69^b^
P05	2.96^a^	12.0^a^	99.1^a^	0.502^a^	1.44^a^	9.08^ab^	0.177^e^	0.404^de^	4.37^b^
P06	2.53^a^	5.98^def^	48.3^de^	0.352^abcd^	0.593^e^	7.46^bc^	0.301^c^	0.646^c^	7.64^b^
P07	2.74^a^	8.23^bc^	65.8^bc^	0.415^abc^	0.901^c^	5.70^c^	0.273^cd^	0.634^c^	6.53^b^
P08	2.70^a^	7.11^bcd^	53.3^bcd^	0.353^abcd^	0.484^e^	4.88^c^	0.231^cde^	0.468^cde^	4.85^b^
P09	2.65^a^	6.47^cdef^	51.2^de^	0.436^a^	0.780^d^	7.22^bc^	0.199^de^	0.486^cde^	4.42^b^
P10	2.29^a^	8.92^b^	68.4^b^	0.266^bcd^	0.590^e^	7.36^bc^	0.179^e^	0.351^e^	5.13^b^
October									
F (*p-value)*	12.16 (≤ 0.05*)	9.56 (≤ 0.05*)	7.59 (≤ 0.05*)	25.76 (≤ 0.05*)	8.85 (≤ 0.05*)	14.22 (≤ 0.05*)	46.62 (≤ 0.05*)	93.63 (≤ 0.05*)	81.96 (≤ 0.05*)
P01	2.21^b^	4.86^c^	36.1^c^	0.192^e^	0.564^b^	3.58^b^	0.213^de^	0.544^bcd^	4.72^bc^
P02	1.57^c^	4.60^c^	35.6^c^	0.211^de^	0.436^b^	2.75^b^	0.315^bc^	0.550^bcd^	4.12^bc^
P03	2.70^ab^	5.89^bc^	49.1^bc^	0.438^a^	1.12^a^	11.5^a^	0.620^a^	1.95^a^	14.5^a^
P04	2.43^ab^	6.65^bc^	50.9^bc^	0.292^bcd^	0.516^b^	3.83^b^	0.389^b^	0.740^b^	5.45^b^
P05	2.84^a^	11.8^a^	93.7^a^	0.494^a^	1.13^a^	7.53^a^	0.223^de^	0.375^cde^	3.42^cd^
P06	2.48^ab^	5.26^bc^	46.7^bc^	0.322^b^	0.310^b^	3.22^b^	0.264^cd^	0.251^e^	2.82^d^
P07	2.52^ab^	7.75^bc^	63.2^ab^	0.312^bc^	0.625^b^	4.00^b^	0.272^cd^	0.546^bcd^	5.98^b^
P08	2.43^ab^	6.45^bc^	50.3^bc^	0.295^bcd^	0.517^b^	3.61^b^	0.254^cd^	0.455^cde^	3.76^bcd^
P09	2.61^ab^	5.80^bc^	49.2^bc^	0.312^bc^	0.415^b^	3.06^b^	0.206^de^	0.327^de^	2.76^d^
P10	2.83^a^	8.43^b^	64.9^ab^	0.225^cde^	0.568^b^	4.58^b^	0.151^e^	0.586^bc^	4.78^bcd^

Mean values for research site (*n* = 5); identical superscripts (a, b, c …) indicated non-significant differences between means within columns (separately for May and October) according to the post-hoc Tukey’s HSD test.

**Fig. 4:**
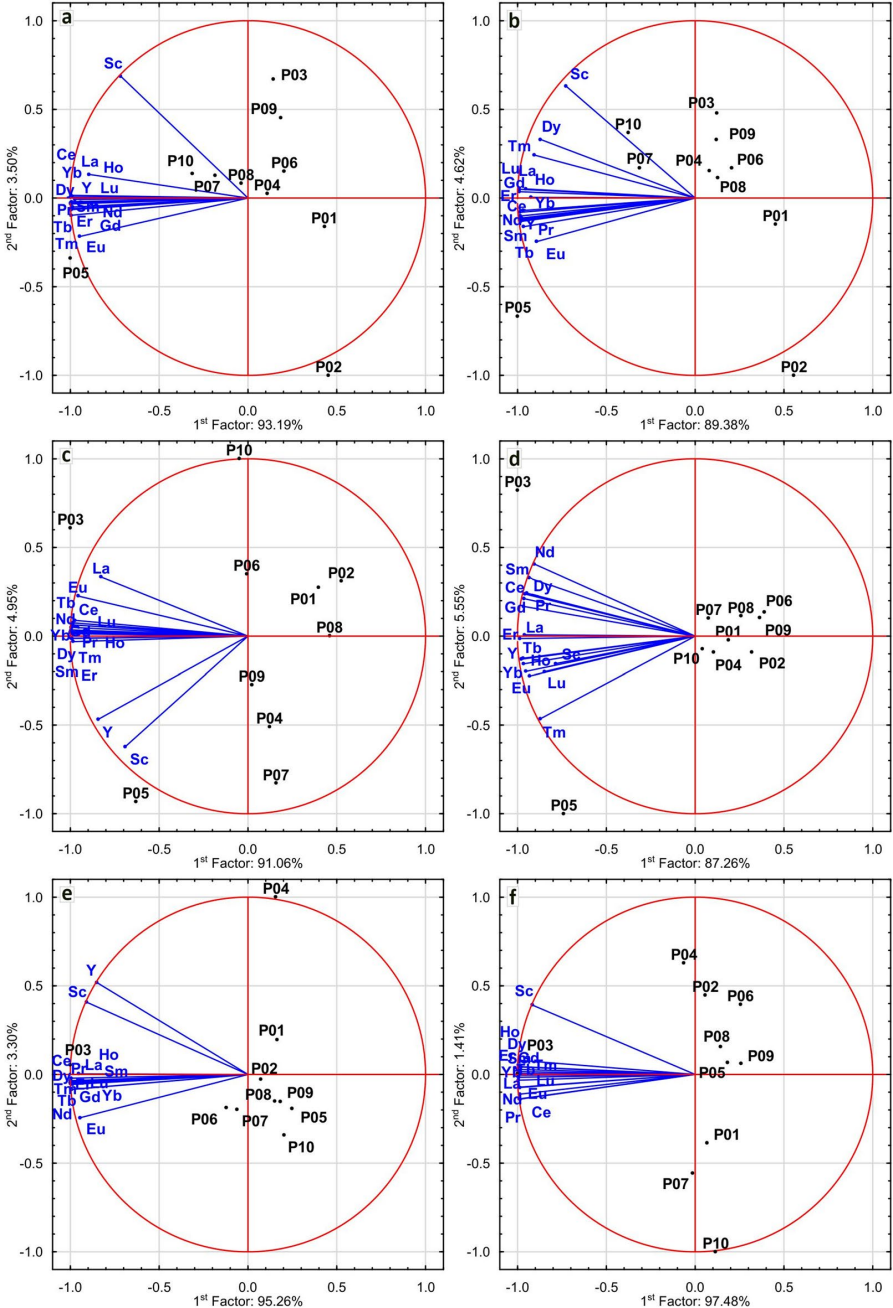
Principal component analysis of Sc, Y and REEs content in soil (**a**,**b**), dandelion roots (**c**,**d**) and leaves (**e**,**f**) in May (**a**,**c**,**e**) and October (**b**,**d**,**f**).

Graphical data presentation by principal component analysis allowed to identify the relationship between elemental content in the soil and the plants, including seasonal variations. The analysis explained a significant part of data variability - more than 90% of variability in all research sites for all materials and seasons. All 14REEs were positively related in all materials and seasons. Scandium and yttrium were positively associated with each other in the roots and leaves of dandelions during spring (May). A weaker association between the variables was observed for plants in autumn (October). Both elements were mostly positively related to rare earth elements. The analysis revealed that research site located in the park (P05), as well as in the low-density residential areas (P07) and in the industrial area (P10), was associated with the content of all analysed elements in the soil in both research seasons. The other research sites, with the exception of P02, were comparable in terms of Sc, Y and 14REEs content in soil for both seasons. The following research sites: the high-density residential areas (P03) and a park (P05) were related to the content of Sc, Y and 14REEs in roots of *T. officinale* in both seasons. Moreover, the sites were more comparable in terms of analysed elemental content in roots in autumn. In terms of content in leaves, as well as in dandelion roots, the research site located in the high-density residential areas (P03) stands out from the others. The analysis also showed that the research sites due to the element content were more comparable in the spring, in contrast to the tendencies observed for roots, or soil (Fig. 4).

## Discussion

Determining environmental concentrations of REEs is difficult due the their high variability across ecosystem^37^. The mean concentration of 14REEs in Polish soils typically ranges from 10 to 100 mg kg^−1^^3^, whereas concentrations in plants are significantly lower^23,26^. Contamination with REEs in soils is commonly linked to smelting and mining. Still, in urban areas, it may result from urbanisation, waste disposal, traffic emissions (e.g., asphalt abrasion, catalytic converters, brake livings)^2,44^, fuel combustion, REE-containing fertilisers, and industrial activity^45,46^. While natural sources define background levels, industrial, transportation, and agricultural sources significantly contribute to the spatial heterogenity in urban soils^6^. Similar tendency might be noticed in our studies, where variability between sites in the city was noted. Due to the lack of heavy industry in Poznań, the major source of REEs contamination in soils might be related to transportation and local agrotechnical and nutrition treatments (green and agricultural areas).

The elevated levels of REEs in urban soils may lead to their uptake and accumulation in plant tissues, particularly in species growing in contaminated zones such as road verges^44^, industrial green belts, arable areas with REE-containing fertilizers application or urban wastelands. Rare earth element content in common plants under natural conditions was usually low^47,48^. Some studies have shown that REEs levels in plants significantly influenced not only by soil concentration but also by anthropogenic inputs in urban areas^10,23^. Research has also confirmed the bioaccumulation and phytoextraction potential of plants in polluted environments, including ferns^49^, herbs and weeds^50^, and native species^51^. However, detailed analyses of bioaccumulation in plants revealed variation in the distribution of REEs in plants. The uptake of REEs by roots from soil to plant is generally low and rarely efficient^47^. This tendency has been documented in some herbaceous plant species - including *Achillea millefolium*, *Trifolium pratense* L., *Festuca arundinacea*,* Sinapis alba* or *Tripleurospermum maritimum*^10,50^, and the findings of the present study on dandelion also confirmed the low bioaccumulation of REEs from soil to plant tissues. This may be related to the chemical form of REEs, as rare earth elements typically occur in soil as ionic compounds, often bound to minerals or organic matter^52^. Furthermore, the fact that rare earth elements are not essential for plant physiology may also contribute to their limited bioconcentration rates^50^. Nevertheless, the content of REEs in the underground organs of plants is higher than in the aboveground organs and might be arranged as follows: root > leaf > stem > flower or fruit^5,48^. This may be due to the possibility that the accumulation rate of REEs from the substrate to the roots is still higher than their translocation rate from the roots to the aerial tissues^47^. However, some research has indicated that this tendency may be different or even opposite, as in the case of our study. The higher content of REEs in the aboveground parts of plants may be related to the location of the research sites, but also to air pollution. In the case of location, one of the factors might be distance from roads, as in a study conducted by Mikołajczak et al.^10^ observed that the content of REEs in leaves and stems of native plant species growing near roads was higher than in roots. Another important factor influencing the REEs in the above-ground parts of plants is the REEs content in atmospheric dust, which can be deposited on the leaves^47^. The importance of distance from local pollution as well as dry and wet deposition was also observed during the present study. The highest concentrations of REEs in leaves were recorded at the site located in a high-density residential area, in close proximity to a car park and lacking significant urban vegetation. In contrast, substantially lower REE levels in above-ground plant parts were observed in a park site characterized by dense vegetation cover. The higher ability to efficiently translocate REEs may also be related to the age of plants. Ding et al.^53^ noted that the content of REEs in young wheat leaves was low and increased in older leaves and stems. At the same time, they deduced that due to the constant total content of REEs in wheat, the source of REEs in the aboveground parts is the REEs accumulated in the roots. Another study has indicated that effective translocation can also be related to some tolerance mechanisms in plants to REEs accumulation. Indeed, in studies conducted with *Dicranopteris linearis*, an increased concentration of REEs was observed in damaged aboveground plant tissues to protect other cells^54^. Moreover, Miao et al.^7^ observed that light rare earth elements can be absorbed and transported to the leaves and stems of plants, particularly those with shorter leaf and stem growth periods. Consequently, damage caused by REEs exposure may be mitigated through transpiration and leaves falling. In our study, we observed a slightly higher ability to translocate efficiently in autumn, which may be related to both the age of the plant and the tolerance mechanism mentioned above. However, in comparison to the findings of Ding et al.^53^, a reduction in the total REEs content in the dandelion was observed, which was most probably also due to maintenance treatments of urban green areas, such as lawn mowing.

The seasonal variation in the content of Sc, Y, and the 14REEs in both soil and plants remained underexplored, although some studies have indicated the occurrence of some fluctuations^16,18,19^. For the soil, seasonal changes in the content of elements studied, are mostly insignificant^19^ or related to the dry or wet period^16^. In our study, we observed a slight decrease in the content of the studied elements in the soil between May and October, which may be related to weather conditions. Previous studies have indicated that one of the sources of REEs in soil may be atmospheric dry and wet depositions^55^. Moreover, higher content of REEs in soil were noticed during dry periods, which is probably due to higher evaporation, less dilution by rainwater, as well as more intense water-rock interaction^16^. Although sampling was carried out after a minimum of three consecutive days without precipitation, the greater cumulative rainfall in the days leading up to the sampling period during the autumn season may have influenced the results. In the case of plants, previous research on the seasonality of REEs is mainly limited to analyzing changes in *Juncus effusus* L., both in natural sites in mountainous areas^18^, and near roads^19^. The study showed that the concentrations of REEs in *J. effusus* shoot vary with the season, especially between May and November. At most of the research sites in natural habitats, an increase in REEs concentrations was observed from May to September, with a significant decrease in November. In addition, one of the sites, which was more exposed to pollution compared to the others, showed maximum REEs content in May and lowest levels in September^18^. On the other hand, studies at sites near roads included the period from April to June confirmed the gradual accumulation of selected elements during the analyzed period for shoots of *J. effusus*. These studies additionally analyzed the REEs content in the roots and observed decreases in the concentration of these elements from April to June^19^. Our research on dandelions has provided further confirmation of previous tendencies - a decrease in content of REEs in the roots and leaves of plants during the autumn period compared to the spring period. There are few studies in the literature that explain this issue, however, based on our observations, we can make some assumptions. The elevated concentration of REEs in the roots during spring may be attributed to an enhanced uptake of REEs from the soil, as evidenced by the higher bioconcentration factor (BCF) observed in May. In addition, tolerance mechanisms in plants to REEs accumulation^7,53^ and the throughfall observed during the autumn period, which may leach atmospheric particles deposited on foliage and of leaf excretion^55^, can also contribute to the reduction of REEs content. Overall, the seasonal changes of REEs accumulation in *T. officinale* are mostly related to the plant’s phenology changes, as well as to the type of land use and agrotechnical activities at green and arable areas.

## Conclusions

The research conducted has proved new findings on human-made pollution caused by urban activities, in the context of the seasonal variation of rare earth elements in soil and plants.

The variation in soil REEs, Sc and Y accumulation was found. The variation of surface soil’s elements accumulation was related to the characteristics of the place.

The translocation of all elements was recorded for almost all sites in both terms of sampling. Our research on dandelions has provided further confirmation of previous tendencies - a decrease in content of REEs in the roots and leaves of plants during the autumn period compared to the spring period. Reduction in the total REEs content in the dandelion was observed. Moreover, we noticed a slightly higher ability to translocate efficiently in autumn.

The presented results may be an important element in managing urban resources and addressing the knowledge gaps in the presence and accumulation of REEs in the urban environment in different seasons. However, it is recognized that they possess certain limitations and do not fully capture all relevant processes, so a more regular monitoring of REEs should be carried out in the future, which would allow the development of better phytoremediation and/or air pollution mitigation strategies in urban areas using herbs or weeds.

## Supplementary Information

Below is the link to the electronic supplementary material.

Supplementary Material 1

## Data Availability

All data will be available from the corresponding author on reasonable request.
