# Supplementary material for: Seasonal variation of rare earth elements in Taraxacum officinale as an indicator of changes in urban pollution
Source: Sci Rep. 2025 Aug 12;15:29496. doi: 10.1038/s41598-025-15371-4 (PMC12343766; doi:10.1038/s41598-025-15371-4)
Supplement: Supplementary file 1 — Supplementary Material 1 [file 41598_2025_15371_MOESM1_ESM.pdf]

Supplementary Table S1. The pH, electric conductivity (EC) and elements' concentrations in soils, roots, and leaves of *Taraxacum officinale*

Parameter	Season	Soil			Roots			Leaves		
		$\bar{x}$±σ	min-max	Q1-Q3	$\bar{x}$±σ	min-max	Q1-Q3	$\bar{x}$±σ	min-max	Q1-Q3
pH	May	7.22±0.46	6.19-7.72	7.21-7.43	-	-	-	-	-	-
	October	7.16±0.35	6.41-7.58	6.99-7.46	-	-	-	-	-	-
EC (mS cm^{-1})	May	0.12±0.04	0.06-0.16	0.11-0.14	-	-	-	-	-	-
	October	0.14±0.03	0.08-0.19	0.11-0.17	-	-	-	-	-	-
Sc (mg kg^{-1})	May	2.58±0.44	1.08-3.15	2.45-2.88	0.368±0.116	0.122-0.579	0.264-0.463	0.312±0.173	0.110-0.795	0.216-0.339
	October	2.46±0.41	1.43-3.32	2.24-2.77	0.309±0.099	0.140-0.577	0.226-0.337	0.291±0.133	0.102-0.662	0.224-0.316
Y (mg kg^{-1})	May	7.11±2.23	4.46-12.4	5.34-7.99	0.788±0.303	0.468-1.58	0.580-0.899	0.639±0.296	0.207-1.35	0.472-0.712
	October	6.75±2.49	3.99-16.7	4.88-7.91	0.620±0.329	0.299-1.65	0.401-0.702	0.634±0.479	0.235-2.24	0.371-0.670
La (mg kg^{-1})	May	11.4±3.95	6.90-24.0	8.77-13.2	1.79±0.652	0.973-3.82	1.30-2.25	1.62±0.722	0.524-3.81	1.33-1.70
	October	11.1±4.15	3.72-26.0	8.35-13.46	0.981±0.474	0.309-2.25	0.552-1.09	1.04±0.735	0.379-3.71	0.596-1.11
Ce (mg kg^{-1})	May	22.7±7.44	13.1-41.9	18.1-24.0	2.73±1.07	1.55-5.98	1.82-3.09	2.56±1.53	0.002-6.85	1.73-2.98
	October	22.2±7.82	10.6-45.9	17.7-24.8	2.12±1.41	0.653-6.27	1.18-2.56	2.28±1.47	0.783-6.89	1.39-2.44
Pr (mg kg^{-1})	May	2.66±0.911	2.08-2.83	2.08-2.83	0.322±0.135	0.182-0.704	0.215-0.363	0.290±0.168	0.012-0.765	0.193-0.337
	October	2.56±1.02	1.97-4.37	2.07-2.81	0.218±0.151	0.075-0.695	0.118-0.259	0.266±0.163	0.090-0.757	0.156-0.294
Nd (mg kg^{-1})	May	10.2±3.55	5.79-19.4	7.98-10.9	1.23±0.515	0.702-2.68	0.806-1.37	1.11±0.638	0.103-2.83	0.756-1.25
	October	9.84±3.76	3.55-24.7	7.84-11.1	0.916±0.607	0.286-3.09	0.529-1.03	0.960±0.637	0.342-2.99	0.577-1.06
Sm (mg kg^{-1})	May	1.99±0.693	1.13-3.80	1.55-2.15	0.233±0.095	0.133-0.487	0.153-0.263	0.207±0.117	0.042-0.546	0.146-0.240
	October	1.87±0.665	0.876-4.13	1.49-2.16	0.163±0.112	0.054-0.667	0.100-0.163	0.177±0.119	0.064-0.561	0.111-0.199
Eu (mg kg^{-1})	May	0.925±0.287	0.576-1.66	0.742-1.02	0.071±0.030	0.038-0.157	0.052-0.079	0.058±0.025	0.001-0.126	0.047-0.060
	October	0.466±0.207	0.227-1.23	0.338-0.531	0.034±0.015	0.014-0.087	0.023-0.042	0.035±0.022	0.015-0.099	0.020-0.038
Gd (mg kg^{-1})	May	2.61±0.953	1.53-5.18	2.02-2.70	0.256±0.102	0.141-0.544	0.168-0.284	0.246±0.129	0.031-0.593	0.175-0.278
	October	2.27±0.941	1.16-5.15	1.58-2.67	0.177±0.132	0.053-0.745	0.097-0.213	0.177±0.119	0.063-0.533	0.104-0.194
Tb (mg kg^{-1})	May	0.333±0.117	0.202-0.642	0.253-0.350	0.032±0.012	0.018-0.066	0.022-0.035	0.028±0.015	0.002-0.067	0.019-0.032
	October	0.289±0.130	0.155-0.767	0.209-0.309	0.020±0.013	0.007-0.073	0.013-0.025	0.023±0.016	0.008-0.070	0.013-0.026
Dy (mg kg^{-1})	May	1.52±0.499	0.937-2.83	1.15-1.62	0.148±0.057	0.081-0.301	0.103-0.164	0.125±0.063	0.023-0.289	0.086-0.137
	October	1.37±0.384	0.788-2.65	1.03-1.63	0.108±0.074	0.033-0.435	0.062-0.135	0.121±0.081	0.041-0.367	0.077-0.131
Ho (mg kg^{-1})	May	0.302±0.097	0.195-0.559	0.232-0.327	0.027±0.010	0.015-0.056	0.019-0.030	0.023±0.011	0.001-0.053	0.016-0.025
	October	0.275±0.091	0.152-0.543	0.199-0.312	0.019±0.011	0.006-0.060	0.012-0.024	0.021±0.015	0.007-0.065	0.014-0.020
Er (mg kg^{-1})	May	0.937±0.284	0.580-1.67	0.752-0.988	0.077±0.029	0.042-0.158	0.054-0.088	0.065±0.030	0.011-0.139	0.047-0.077
	October	0.794±0.288	0.437-1.79	0.582-0.922	0.055±0.027	0.018-0.129	0.036-0.070	0.059±0.043	0.021-0.181	0.034-0.059
Tm (mg kg^{-1})	May	0.130±0.037	0.078-0.232	0.103-0.144	0.010±0.004	0.005-0.020	0.007-0.011	0.008±0.004	0.001-0.018	0.006-0.009
	October	0.107±0.033	0.057-0.195	0.076-0.126	0.006±0.004	0.002-0.022	0.004-0.008	0.008±0.006	0.003-0.024	0.005-0.008
Yb (mg kg^{-1})	May	0.884±0.250	0.547-1.53	0.677-0.984	0.058±0.021	0.032-0.122	0.041-0.065	0.049±0.023	0.011-0.112	0.035-0.057
	October	0.745±0.314	0.386-1.96	0.539-0.837	0.042±0.019	0.015-0.101	0.026-0.054	0.051±0.037	0.018-0.163	0.029-0.056
Lu (mg kg^{-1})	May	0.136±0.037	0.081-0.229	0.108-0.152	0.008±0.003	0.004-0.018	0.006-0.009	0.007±0.003	0.001-0.015	0.005-0.008
	October	0.101±0.035	0.053-0.216	0.075-0.110	0.007±0.004	0.002-0.020	0.004-0.008	0.007±0.005	0.003-0.023	0.004-0.008

Supplementary Table S2. Mean value for contamination factor (CF) and pollution load index (PLI) at research sites, where highlighted values indicated moderate contamination factor (MCF)

Element	Season	Research site									
		**P01**	**P02**	**P03**	**P04**	**P05**	**P06**	**P07**	**P08**	**P09**	**P10**
Sc	May	0.264	0.185	0.298	0.277	0.325	0.278	0.301	0.297	0.291	0.321
	October	0.243	0.172	0.297	0.267	0.312	0.272	0.276	0.267	0.286	0.311
Y	May	0.216	0.211	0.263	0.297	0.530	0.263	0.363	0.313	0.285	0.393
	October	0.214	0.202	0.260	0.293	0.520	0.232	0.342	0.284	0.255	0.371
La	May	0.294	0.305	0.402	0.382	0.731	0.356	0.515	0.381	0.542	0.504
	October	0.285	0.290	0.394	0.370	0.711	0.340	0.504	0.379	0.518	0.481
Ce	May	0.295	0.286	0.423	0.397	0.763	0.383	0.509	0.411	0.357	0.525
	October	0.285	0.275	0.389	0.420	0.747	0.383	0.497	0.405	0.346	0.513
Pr	May	0.303	0.295	0.418	0.403	0.789	0.374	0.516	0.414	0.361	0.541
	October	0.255	0.289	0.381	0.399	0.787	0.367	0.489	0.386	0.361	0.531
Nd	May	0.311	0.305	0.417	0.418	0.815	0.381	0.531	0.427	0.369	0.561
	October	0.283	0.292	0.394	0.414	0.812	0.378	0.497	0.421	0.363	0.542
Sm	May	0.319	0.307	0.427	0.428	0.842	0.405	0.519	0.443	0.378	0.575
	October	0.286	0.291	0.422	0.422	0.784	0.376	0.505	0.402	0.357	0.521
Eu	May	0.812	0.841	0.829	0.883	**1.047**	0.887	0.982	0.991	0.821	**1.006**
	October	0.454	0.498	0.462	0.441	0.862	0.449	0.641	0.434	0.477	0.757
Gd	May	0.414	0.422	0.548	0.550	**1.103**	0.506	0.708	0.635	0.506	0.783
	October	0.388	0.306	0.463	0.503	0.888	0.499	0.695	0.491	0.471	0.704
Tb	May	0.355	0.357	0.450	0.476	0.954	0.440	0.604	0.513	0.423	0.649
	October	0.322	0.309	0.329	0.382	0.832	0.383	0.518	0.427	0.408	0.616
Dy	May	0.297	0.291	0.377	0.405	0.745	0.357	0.484	0.413	0.359	0.521
	October	0.267	0.266	0.367	0.362	0.476	0.323	0.471	0.380	0.386	0.519
Ho	May	0.297	0.289	0.359	0.403	0.732	0.370	0.473	0.425	0.366	0.511
	October	0.286	0.257	0.333	0.392	0.580	0.342	0.467	0.364	0.356	0.462
Er	May	0.317	0.334	0.386	0.426	0.772	0.378	0.490	0.450	0.378	0.530
	October	0.300	0.259	0.321	0.337	0.628	0.325	0.486	0.349	0.346	0.430
Tm	May	0.285	0.310	0.362	0.403	0.678	0.357	0.453	0.468	0.358	0.495
	October	0.239	0.241	0.335	0.345	0.443	0.332	0.449	0.311	0.306	0.416
Yb	May	0.291	0.298	0.372	0.407	0.685	0.371	0.453	0.502	0.361	0.490
	October	0.267	0.221	0.344	0.347	0.619	0.333	0.439	0.311	0.304	0.382
Lu	May	0.300	0.324	0.385	0.413	0.690	0.391	0.484	0.565	0.384	0.508
	October	0.270	0.207	0.341	0.350	0.508	0.271	0.359	0.299	0.296	0.368
PLI – 14REEs	May	0.000	0.000	0.000	0.000	0.006	0.000	0.000	0.000	0.000	0.001
	October	0.000	0.000	0.000	0.000	0.002	0.000	0.000	0.000	0.000	0.000
